# Supplementary material for: Real-time forecasting of COVID-19-related hospital strain in France using a non-Markovian mechanistic model
Source: PLoS Comput Biol. 2024 May 17;20(5):e1012124. doi: 10.1371/journal.pcbi.1012124 (PMC11139328; doi:10.1371/journal.pcbi.1012124)
Supplement: S1 Text — (PDF) [file pcbi.1012124.s001.pdf]

Technical details for real-time forecasting of COVID-19 in France using a non-Markovian mechanistic model

S1 Text

Alexander Massey*, Corentin Boennec, Claudia Ximena Restrepo-Ortiz,

Christophe Blanchet, Samuel Alizon, Mircea T. Sofonea

* corresponding author: stats.massey@gmail.com

May 10, 2024

This document provides further details and mathematical formulations to sections as presented in the publication with the aforementioned title found in the PLOS Computational Biology. Sections in the supplement generally correspond to the same section titles of the main article.

COVIDici notation tables

notation	meaning
S_i, S_i^{V}	unvaccinated, vaccinated susceptible individuals of age class i
$J_{i,\tau}, J_{i,\tau}^{\mathrm{V}}$	unvaccinated, vaccinated non critical infectious individuals of age class i on their τ-th day of infection
$Y_{i,\tau}, Y_{i,\tau}^{\mathrm{V}}$	unvaccinated, vaccinated critical infectious individuals of age class i on their τ-th day of infection
$H_{i,\tau}$	long-stay ICU patients of age class i on their τ-th hospitalisation day
$W_{i,\tau}$	other critical hospitalised patients of age class i on their τ-th hospitalisation day
R_i	recovered immunised individuals of age class i
D_i	deceased individuals of age class i

Table A – **Main clinical-epidemiological compartments/densities simulated by the `COVIDici` model.** (NB: these quantities vary across calendar time t, scales and location ℓ – which are implicit here for the sake of readability).

notation	meaning	national prior	sub-national prior	ref.
$\mathcal{R}_0$	basic reproduction number	$\mathcal{U}\,[2.39, 3.61]$	only inferred at national level	[1]
$\mathbb{E}\,[\mathrm{H}]$	contamination-to-ICU admission delay expectation (days)	$\mathcal{U}\,[10.7, 17.7]$		
σ_{H}	contamination-to-ICU admission delay standard deviation (days)	$\mathcal{U}\,[3.28, 5.59]$		
t_0	initiation day (YY-MM-DD)	2020-01-$\mathcal{U}$[15,24]	2020-$\mathcal{U}$[01-10,03-10]	
$\mathcal{C}\,(t)$	transmission factor at calendar day t	$\mathcal{U}\,[0.4, 1]$ or computed (for the last 21 days, see appendix)		
$\mathbb{E}\,[\mathrm{P}]$	long ICU stay length expectation (days)	$\mathcal{U}\,[12.7, 19.0]$	$\mathcal{U}\widehat{\mathbb{E}\,[\mathrm{P}]}^{\mathrm{nat}} \times [0.9, 1.1]$	
$\mathbb{E}\,[\Upsilon]$	non long-stay ICU admission-to-death delay (days)	$\mathcal{U}\,[7.2, 10.8]$	$\mathcal{U}\widehat{\mathbb{E}\,[\Upsilon]}^{\mathrm{nat}} \times [0.9, 1.1]$	
$\mathfrak{C}_{\mathrm{F}}$	infection fatality ratio correction factor	$\mathcal{U}\,[0.56, 1.20]$	$\mathcal{U}\widehat{\mathbb{E}\,[\mathfrak{C}_{\mathrm{F}}]}^{\mathrm{nat}} \times [0.9, 1.1]$	
$\mathfrak{C}_{\mathrm{M}}$	long-stay ICU fatality ratio correction factor	$\mathcal{U}\,[0.23, 1.24]$	$\mathcal{U}\widehat{\mathbb{E}\,[\mathfrak{C}_{\mathrm{M}}]}^{\mathrm{nat}} \times [0.9, 1.1]$	
$\mathfrak{C}_{\Psi}$	long-stay ICU frequency correction factor	$\mathcal{U}\,[0.83, 1.26]$	$\mathcal{U}\widehat{\mathbb{E}\,[\mathfrak{C}_{\Psi}]}^{\mathrm{nat}} \times [0.9, 1.1]$	

Table B – **Key parameters inferred by the `COVIDici` model.**
The national priors were provided by the fitting/inference procedure of the `COVIDSIM` − `FR` model, according to [1]. (NB: these quantities vary across calendar time t, scales and location ℓ – which are implicit here for the sake of readability).

notation	meaning	value(s)	ref.
$\Lambda_{i,\ell}(t)$	force of infection experienced by individuals of age class i, in location ℓ, on calendar day t	computed (see main text)	[1]
S°_{ℓ}	population size in location ℓ	publicly available demographic data (Insee database)	[2]
$c_{i,\ell}(t)$	per capita contact ratio of age class i, in location ℓ, on calendar day t	inferred or computed within $\mathcal{C}_{\ell}(t)$	[1]
$q_{\ell}(t)$	viral evolution-induced updating factor of the reproduction number at calendar day t, in location ℓ		[3–5]
θ_i	critical illness probability for individuals in age class i upon infection	computed from the age-stratified IFR and hospital flows profiles, as in [1]	[1, 6–8]
ψ_i	long-stay ICU admission probability of critically ill patients in age class i upon hospitalisation		
μ_i	long-stay ICU fatality ratio for age class i		
δ_i	daily (first-dose) vaccination rate for age class i	publicly available time series (linearly extrapolated from last 21 days)	[9]
ν_{C}	vaccination-induced critical illness reduction factor	0.1	[10]
$\nu_{\mathrm{I}}, \nu_{\mathrm{T}}$	vaccination-induced infection, infectivity reduction factor	0.2	
$a_{\ell,t}$	notified number of ICU admitted patients in location ℓ on day t	publicly available time series (SI-VIC database)	[11]
$a^{\circ}_{\ell,t}$	Gaussian rounded 7-day moving-averaged number of admitted patients in location ℓ on day t	computed from $a_{\ell,t}$ (see main text)	
$a^{\mathrm{s}}_{\ell,t}(\mathbf{x})$	number of ICU admitted patients in location ℓ on day t simulated by `COVIDici` being set with parameter set $\mathbf{x}$		
$\mathcal{L}_{\ell,\mathcal{T}}(\mathbf{x})$	likelihood of parameter set $\mathbf{x}$ with respect to the daily ICU admission times series in location ℓ, over time window $\mathcal{T}$	computed from $a^{\circ}_{\ell,t}$ and $a^{\mathrm{s}}_{\ell,t}(\mathbf{x})$ (see main text)	

Table C – **Main constrained quantities of the `COVIDici` model.**

notation	meaning	value(s)	ref.
Z	generation time random variable (NB: its support is $\mathrm{Z}(\Omega) = [\![1, \tau_{\mathrm{Z}}^{\max} := 11]\!]$ days)	Weibull distribution with mean 4.8 days and variance 5.3 days^2	[12]
$\zeta_{\cdot}$	generation time probability mass function	computed from the distribution of Z	[1]
H	contamination-to-ICU admission delay random variable	Weibull distribution with mean $\widehat{\mathbb{E}[\mathrm{H}]}$ and variance $\widehat{\sigma_{\mathrm{H}}^2}$	
η_τ	ICU admission probability on the τ-th day of infection	computed from the distribution of H	
P	long ICU stay length random variable	$\mathcal{E}\left(\widehat{\mathbb{E}[\mathrm{P}]}^{-1}\right)$	
ρ_τ	ICU discharge or death probability on the τ-th day of hospitalisation	computed from the distribution of P	
Υ	non long-stay ICU admission-to-death delay random variable	$\mathcal{E}\left(\widehat{\mathbb{E}[\Upsilon]}^{-1}\right)$	
υ_τ	non long-stay ICU death probability of critical patients on their τ-th day of hospitalisation	computed from the distribution of Υ	

Table D – **Main distributions involved in the `COVIDici` model.**

notation	meaning
$\mathcal{A}$	set of age classes according to [9] $([0,4], [5,9], [10,11], [12-17], [18-24], [25-29], [30-39], [40-49], [50-59], [60-69], [70-74], [75-79], [80,\infty)$ years old)
$\lfloor \cdot \rceil$	Gaussian (half-to-even) rounding
$\mathcal{U}[a,b]$	continuous uniform distribution on $[a,b]$
$\mathcal{E}(\lambda)$	exponential distribution with rate λ
$\widehat{\cdot}$	statistical estimation ($\cdot^{\mathrm{nat}} \equiv$ at national level)
$\varrho_\ell^{\mathrm{s}}(t)$	simulated recovery ratio in location ℓ at calendar day t
$\iota_\ell^{\mathrm{s}}(t)$	simulated immunisation ratio in location ℓ at calendar day t

Table E – **Additional notation table.**

Transmission factor projection

The delay between contamination and ICU admissions induces a lag between community dynamics and ICU time series. In the meantime, by construction, the inference procedure works well when the key parameters are stationary over several weeks but is less reactive for capturing recent changes. In order to update the simulated community transmission with the early shifts appearing in the ICU time series, we imposed the current transmission factor $\mathcal{C}(t)$, which modulates the force of infection for the forecasting time window, to be computed for a given location ℓ from the the last day of available ICU admission data t,

$$\widehat{\mathcal{C}_{\ell}(t)} := \frac{\widehat{\mathcal{R}_t}\left(\left(a^{\circ}_{\ell,\tau}\right)_{\tau\in[t-20,t]}\right)}{\left(1-\iota^{\mathrm{s}}_{\ell}(t)\right)\widehat{\mathcal{R}_0}^{\mathrm{nat}}}, \tag{1}$$

where $\widehat{\mathcal{R}_t}\left(\left(a^{\circ}_{\ell,\tau}\right)_{\tau\in[t-20,t]}\right)$ is the median temporal (or effective) reproduction number, estimated by the standard `EpiEstim` procedure [13, 14] over the last three weeks of daily ICU admissions data (pre-treated as for the likelihood computations, see main text), parametrised with the discretised serial interval distribution $\zeta_{\cdot}$ from [12]. $\widehat{\mathcal{R}_0}^{\mathrm{nat}}$ stands for the median basic reproduction number estimated by the MCMC procedure performed at the national level. Finally, $\iota^{\mathrm{s}}_{\ell}(t)$ is the local and current immunisation ratio simulated by the model, the computation of which will be exposed afterwards.

The derivation of Equation (1) is given by the following special case. Let us assume that there is a single, unvaccinated infectious case in location ℓ at calendar day t, and that, formally, its generation time is concentrated in that day (let us say $\zeta_1 = 1$). Therefore, the probability of infection for a individual from age class i in location ℓ at calendar day t is given by the discrete-time force of infection, according to equation

$$\Lambda_{i,\ell}(t) = \frac{1}{1+\frac{S^{\circ}_{\ell}}{\mathcal{R}_0\mathcal{C}_{\ell}(t)}} \simeq \frac{\mathcal{R}_0\mathcal{C}_{\ell}(t)}{S^{\circ}_{\ell}}, \tag{2}$$

which is a very good approximation since $\mathcal{R}_0\mathcal{C}_{\ell}(t) < 10$ while $S^{\circ}_{\ell} > 7.5\times10^4$ inhabitants (for the least inhabited French department, namely Lozère). Meanwhile, the same probability can be seen, under the mean-field assumption, as a Bernoulli trial consisting of being part of the $\mathcal{R}_{\ell,t}$ secondary cases drawn

among the individuals still non immunised at time t $(1 - \iota_\ell(t))\, S_\ell^\circ$, that is

$$\Lambda_{i,\ell}(t) = \frac{\mathcal{R}_{\ell,t}}{(1 - \iota_\ell(t))\, S_\ell^\circ}. \tag{3}$$

Equation (1) is then straightforward from combining Equations (2) and (3), and replacing the key quantities with their estimates.

Finally, the simulated immunisation ratio requires first to calculate the simulated recovered ratio, being the fraction of local population that has recovered from a SARS-CoV-2 infection – which we assumed provide a perfect immunity for the investigated time window,

$$\varrho_\ell^{\mathrm{s}}(t) := 1 - \frac{S_\ell(t) + S_\ell^{\mathrm{V}}(t)}{S_\ell^\circ - D_\ell(t)}, \tag{4}$$

then the simulated immunisation ratio is obtained by adding to the previous quantity the fraction of non-recovered invididuals that are vaccinated and effectively protected from infection (which, from a polarised/all-or-nothing viewpoint, occurs with probability $1 - \nu_{\mathrm{I}}$),

$$\iota_\ell^{\mathrm{s}}(t) := \varrho_\ell^{\mathrm{s}}(t) + (1 - \varrho_\ell^{\mathrm{s}}(t))\, \frac{S_\ell^{\mathrm{V}}(t)\,(1 - \nu_{\mathrm{I}})}{S_\ell^\circ - D_\ell(t)}. \tag{5}$$

Parameter inference

Parameters were then inferred under the Bayesian framework by running a Markov chain Monte Carlo (MCMC) algorithm – implemented in the `BayesianTools R` package [15] – over a set of 12,000 realisations of the model. The last 2,000 iterations were used to generate the median and the 95% credibility interval for the parameters and 95% forecasting range for the time series, assuming a Poisson likelihood for daily ICU admissions. Except for the reproduction number $\mathcal{R}_0$, the expectation and the variance of the infection-to-hospitalisation delay, which were inferred at the nationwide level only, all the parameters were independently fitted for each sub-national administrative division.

Summary values of the main simulated parameters of interest (e.g. ICU admissions, ICU occu-

pancy, etc.) were originally saved using only the mean and the two quantiles $q_{2.5}$ and $q_{97.5}$. However, it has become commonplace in forecast hub projects (i.e. [16]) to consider as many as 23 quantiles in order to assess the entire predictive distribution at a small granular level. For the retrospective evaluation, this necessitated the imputation of missing quantiles that were not saved under the standard COVIDici workflow in order to conduct a similar comparison. As a result, we fit a skewed normal distribution when the point forecast value was greater than 6 (daily events) or a log-normal distribution when the point was less than 6 (i.e. close to zero) using quantile matching. Fitting the log-normal distribution when the number of daily events was less than or equal to 6 forced all the quantile forecasts to be non-negative, which can technically occur when fitting skewed normal distributions. Code and documentation for this post-processing step is available in our public git repository https://gitlab.in2p3.fr/ete/covidici_public/.

Deployment workflow

The first step in the updating phase was the upload of the vaccination, critical care, and mortality data every evening after it had been updated by Santé Publique France [17–20]. During the night, a job was assigned to each territory that creates a workspace on a node of the cluster, which contained all the data and scripts necessary to derive all the parameters and created time series of each type of observable data on the COVIDici application (ICU admissions, ICU occupancy, etc.). The outputs were generated as CSV files and repatriated to the same cluster in a git project. This was followed by an update of the git project on the project server, which contained the code running the interface and all the data used by it. In the morning, the Shiny application was updated by the Institut Français de Bioinformatique (IFB) and made available at: https://cloudapps.france-bioinformatique.fr/covidici/.

Benchmarking

Criteria for inclusion

Although COVIDici updated its model and public app interface daily, we adopted the format used by the European COVID-19 Forecast Hub to construct our retrospective evaluation for consistency. Therefore, we recovered historical git submissions made on Sundays (or at the latest Mondays in two cases) between January 30, 2021, to December 2, 2021, the day of the first detected infection from the Omicron variant in France. We then extracted weekly forecasts for the 1- to 4-week horizons which corresponded to target forecast dates always falling on subsequent Saturdays. The last day of available hospital data used to update COVIDici usually corresponded to the day before the submission and as early as the previous Thursday, depending on the data publication delay for a given region. A few of the weeks had no submissions because of server maintenance to the cluster. These delays, as well as the occasional revision of official estimates, could not be avoided and represent some practical aspects of real-time forecasting.

Original estimates were produced and presented on a daily basis using rolling averages to smooth any weekly effect. Evaluating forecasts for hospital demand on a daily basis is problematic because the actual number of hospital admissions on a given day tends to be systematically under-reported on weekends and over-reported during the beginning of the following work week. This data artefact, known as weekly seasonality, leads to a discrepancy between actual hospital demand (i.e. what we want to know but is unobservable) and the figures officially reported by state agencies. We presented our forecasts on a daily level because increased granularity is more useful from a planning perspective, including the potential impact of non-pharmaceutical interventions known to be implemented on a given day. However, forecasts were evaluated on a weekly basis to limit the weekly seasonality and because collaborative forecasting projects such as the European and US Covid-19 Forecast Hubs already use weekly evaluations.

Target variable

Since COVIDici is based on a mechanistic model, it provided daily updates of coherent estimates for many epidemiological indicators of interest such as daily and cumulative mortality, daily and cumulative infections, current infections, ICU admissions, and temporal reproduction number at the French national, regional and departmental levels. For the evaluation, we only focus on forecasts of ICU occupancy because it is the most relevant indicator of hospital strain available in the SI-VIC database. Readers wishing to download this hospital data for themselves are encouraged to consult ./scripts/update_master.R in [21] to find the relevant URLs to download directly from SI-VIC.

Baseline models

Baseline models were evaluated based on a rolling forecasting origin (with non-fixed window length) starting on August 2, 2020. It should be noted that several other baselines were originally considered, e.g. auto-regressive (AR) models using various boosting techniques for variable selection potentially with predictors derived from testing data, and are still available in the provided code [21]. Ultimately, since predictor variables themselves need to be forecasted in the future and the expected delay between positive testing is not longer than 2 weeks, the following three baseline models were selected in order to simplify the presentation of the results:

- **ETS+ARIMA** is an ensemble of an ARIMA and an exponential smoothing (ETS) model fit. It uses a log transformation of the rolling 7-day average of the ICU occupancy up until the last day of data available to COVIDici for each week matching the inclusion criteria. The ensemble was implemented using the fable package in R with the default settings to automatically identify model parameters for the 43 weeks of the evaluation period and 14 national and sub-national geographic areas. ARIMA requires that the time series is stationary which was automatically detected with package defaults using differencing. Prediction intervals were simulated by sampling from the residuals assuming a normal distribution. This baseline is consistent with classical statistical modelling approaches for time series without the inclusion of external covariates. Mathematical de-

tails and a full tutorial regarding the implementation of these models are available at [22]. Code implementing the ensembling procedure is available in ./scripts/classical_forecasts.R of the git repository.

- **AR-Lasso** is an AR machine learning type model implemented using the caretForecast package. This model uses a Box-Cox transformation to stabilise the variance and a linear regression for the past 21 lags as well as Fourier terms to account for potential weekly seasonality. It performs variable selection and regularisation using least absolute shrinkage and selection operator (Lasso) to optimise predictive performance. Formally, the Lasso coefficient vector, $\beta \in \mathbb{R}^p$, minimises the following loss function:

$$(Y - X\beta)^T(Y - X\beta) + \lambda|\beta|_1 \tag{6}$$

where $|\beta|_1 = \sum_{j=1}^{p} |\beta|_j$, $Y \in \mathbb{R}^n$ is the time series of the target variable (e.g. ICU occupancy), $X \in \mathbb{R}^{n \times p}$ is the design matrix containing relevant lags of the target variable and Fourier terms for seasonality and λ is the tuning parameter optimised using time series cross-validation as described by [22] with a 14-day cross-validation horizon. Predictions intervals were recursively simulated using a bootstrap of the one-step ahead residuals from the training set, summarised into predicted quantiles, and then smoothed with a 7-day rolling average to be compared to COVIDici. Thus the seasonality was accounted for in the model and then removed afterwards. This model is included mostly as a cautionary example of how certain evaluation metrics can be misleading. In practice, it avoids large errors by avoiding large predictions at peaks of the waves, which is an undesirable behaviour.

- **Naive** is a baseline and a special case of an AR-1 where the first lag is an offset. It was also implemented using the fable package. The point forecast is simply the last observed value and is optimal if the time series is a random walk (i.e. unpredictable white noise). Essentially this model is the best choice if the modeller believes that there is no exploitable structure in the time series to be used in a prediction. Note that the naive model will always have at least one (near) perfect prediction

for a given forecast horizon towards the top of waves because it will catch the trailing side of the wave as the numbers inevitably decrease after the peak. This produces an artifact in evaluation metrics that are scaled using the naive model because this tends to occur at a time when other more reasonable forecasters overshoot the top of the wave producing particularly abnormally bad evaluation scores.

Metrics

Standard metrics

Both the US and European COVID-19 Forecast Hubs recommend using the absolute error (AE) to evaluate the point forecast, and the empirical coverage rate to evaluate individual prediction levels or the weighted interval score (WIS) to assess the forecast distribution as a whole. The WIS is a proper scoring rule that generalises the absolute error and gives penalties for interval spread as well as for over- and under-prediction. Formally speaking, for the true value of the target variable y, we have a forecast distribution F with median m that contains a set of K prediction intervals whose respective upper (u) and lower (l) limits are the $\frac{\alpha}{2}$ and $1-\frac{\alpha}{2}$ quantiles of the F. WIS is defined as the following weighted sum:

$$WIS = \frac{1}{K+0.5}(0.5\cdot \mid y-m \mid + \sum_{k=1}^{K} \frac{\alpha_k}{2} \cdot IS_{\alpha_k}(F,y) \tag{7}$$

where for a single interval k, the interval score is computed as

$$IS_{\alpha_k}(F,y) = (u-l) + \frac{2}{\alpha_k}(l-y)\cdot \mathbb{1}(y \leq l) + \frac{2}{\alpha_k}(y-u)\cdot \mathbb{1}(y \geq u) \tag{8}$$

with $\mathbb{1}$ being an indicator function and α_k the nominal coverage of interval k. Note that if only the median is included (i.e. $K = 0$) then the WIS simplifies to the AE. Furthermore, as the number of equally spaced intervals increases, the WIS converges to the Continuous Ranked Probability Score. We refer readers to [23] for a deeper technical explanation and [24] for details regarding implementation.

Limitations of standard metrics

When aggregating AE or WIS over geographic units (e.g. regions) we can use the mean as a summary function. However, many of the forecasters (including COVIDici) tend to greatly over-predict the peaks of waves, which produces outlier errors that can distort the interpretation of mean WIS or mean AE when aggregating over time. Using the median could help mitigate against the effects of these outliers, but this has the drawback of representing typical forecast errors that likely occur between the waves rather than at their peaks, which somewhat defeats the purpose of evaluating the surveillance system whose primary task is to warn of future capacity overloads.

Effectively evaluating forecasters at the wave peaks is further complicated by the fact that these over-predictions might be (at least partially) explained by survivor bias that occurs every time a non-pharmaceutical government intervention is implemented. For example, in COVIDici, we predict the future outcome that would occur assuming no change in the transmission pattern (meaning no intervention and no spontaneous behavioural change), but for the evaluation we only observe the future outcome that occurred with the intervention.

Figure A illustrates this notion of survival bias. The dashed line is the counterfactual ICU occupancy that will hypothetically occur without intervention, i.e. what we want to estimate. The black line is the observed ICU occupancy that we observe (i.e. with potential interventions). The shaded area is the undeserved absolute error we would see due to survivor bias if we had a perfect prediction of the dashed line. Conversely, if we had a perfect prediction of the "observed" ICU occupancy, then our absolute error would be scored as zero despite the "true absolute error" being the shaded region. Furthermore, metrics related to the forecast distribution, i.e. the coverage rate and WIS, could also be substantially affected by this bias.

Binary metrics

To mitigate the aforementioned limitations, we introduce an additional evaluation metric that is robust against the negative effects of survival bias. We do this by evaluating forecasts with arbitrary thresholds

Figure A – **Artistic representation of the survivor bias that may occur after a restrictive governmental intervention.**

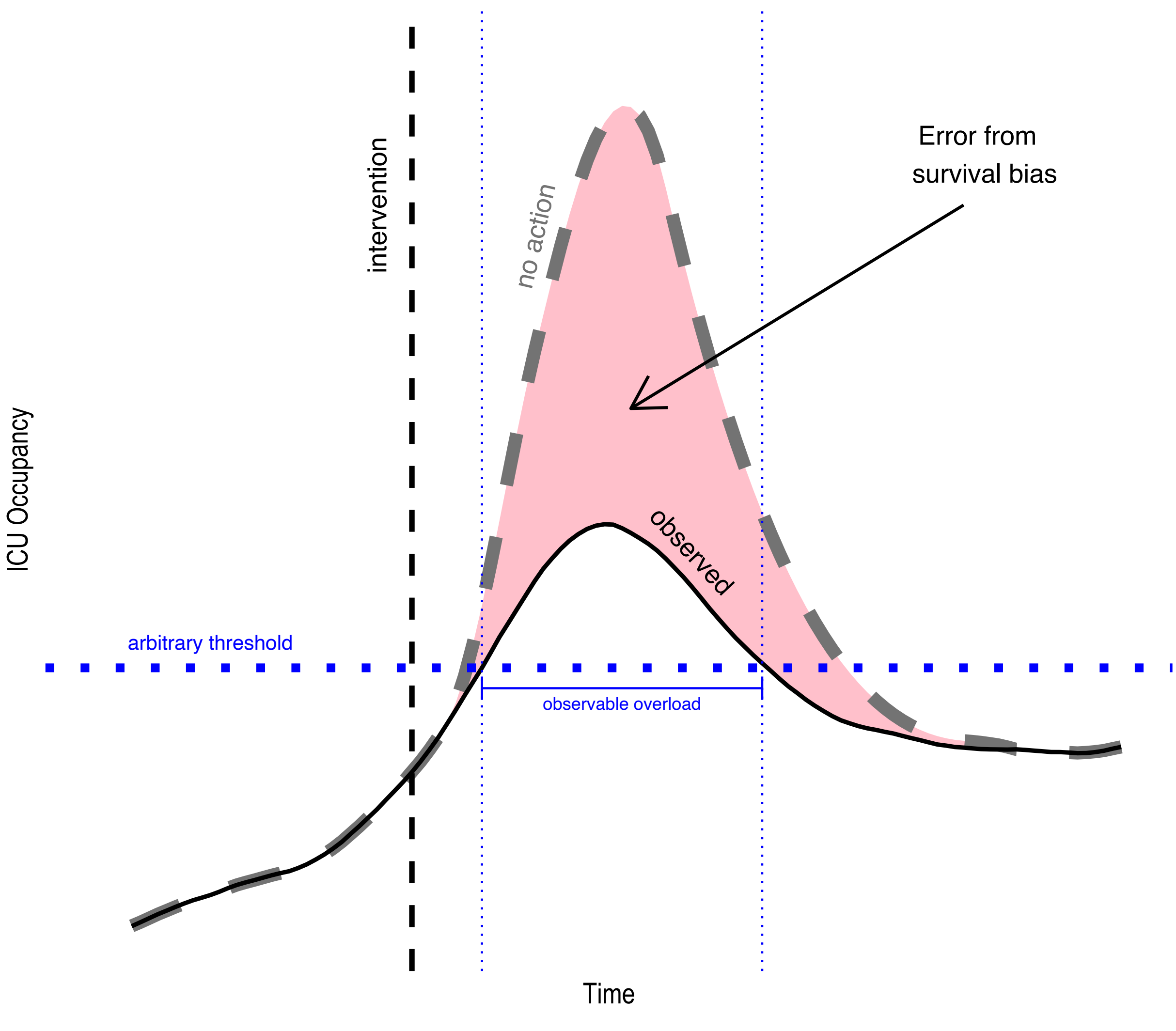

and converting the target variable into a binary variable representing ICU overload and underload. To allow for comparability between geographical units with different population sizes, we defined the threshold as the percentage of the ICU occupancy observed in that geographical unit in the first wave in 2020.

One logical metric is correct prediction rate given that overload was observed, which is also known as the sensitivity. In Figure A we see that in areas of observable overload defined with respect to an arbitrary threshold, the dashed line that we want to predict will always predict overload as well under the very reasonable assumption that government interventions intended to reduce hospital strain do not increase it. This means that forecasters, such as COVIDici, attempting to predict the dashed line will not be unfairly penalised for forecasting what they are intended to.

Computing the specificity of our estimates, i.e. the correct prediction rate given that underload was observed, is important to identify models that predict overload too often. We note that the same argument for robustness does not hold for areas of observable underload but emphasise that errors during these periods are not expected to be as severe.

A proper scoring rule for binary forecasts is the Brier score (BS) which is defined as:

$$BS = \frac{1}{N}\sum_{n=1}^{N}(f_n - y_n)^2 \tag{9}$$

where f_n is the predicted probability of overload event $y_n \in \{0, 1\}$ with $n = 1, ..., N$ denoting all the events in the scope of the evaluation. The predicted probability f_n can be approximately calculated as

$$f_n \approx 1 - \min\{l \mid l \geq T\} \tag{10}$$

where l denotes the collection of lower bounds of the forecast intervals and T the arbitrary threshold.

Bias-Corrected and Accelerated (BCa) Confidence Intervals (CIs)

Let us consider the comparison of two arbitrary models indexed by $k \in a, b$ based on their respective summary metrics denoted as S_k. S_k is a function of the ground truth vector $y := (y_1, y_2, \ldots, y_t)^T$ and the forecast $\hat{y}_k := (\hat{y}_{k,1}, \hat{y}_{k,2}, \ldots, \hat{y}_{k,t})^T$ where $\hat{y}_{k,t}$ is forecast of model k on forecast date $t = 1, 2, \ldots, T$ and T is total number of forecast dates common to both models. For example, within metropolitan France at the four-week horizon we may want to compare the median AE for COVIDici, denoted $S_a(\hat{y}_a, y)$, using the naive baseline model, denoted $S_b(\hat{y}_b, y)$. The population parameter of interest θ is defined as $\theta := \frac{S_a(\hat{y}_a, y)}{S_b(\hat{y}_b, y)}$ which can be used on the log scale if desired. We want to test the null hypothesis $H_0 : \theta = 1$ (or $H_0 : \theta = 0$ if log scale is used), which are both equivalent to $H_0 : S_a(\hat{y}_a, y) = S_b(\hat{y}_b, y)$. In our example we are only aggregating over time, so we want to resample from $X_1, X_2, \ldots, X_t$ where $X_t := (\hat{y}_{a,t}, \hat{y}_{b,t}, y_t)^T$ is the triple containing the ground truth and forecasts for models a and b respectively at forecast date t. Note that if we aggregate over both space and time then t is an index specific to both forecast date and geographic region. The non-parametric bootstrap routine with BCa CIs is as follows:

1. **Bootstrap Sample Generation:** Generate R bootstrap samples denoted as $X_1^*, X_2^*, \ldots, X_t^*$, by randomly sampling with replacement T times from the observed data $X_1, X_2, \ldots, X_t$.

2. **Parameter Estimation:** Compute the sample statistic of interest for each bootstrap sample, denoted as $\hat{\theta}_r^*$ for $r \in 1, 2, \ldots, R$.

3. **Compute Empirical Bias Estimate:** This is the proportion of bootstrap replicate statistics less than the observed statistic $\hat{\theta}$.
$$\hat{b} = \frac{\#\left\{\hat{\theta}_r^* < \hat{\theta}\right\}}{R}$$

4. **Compute Acceleration Parameter:** Using a finite-sample Jackknife procedure, the acceleration constant $\hat{a}$ is given by:
$$\hat{a} = \frac{\sum_{t=1}^{T}(\hat{\theta}_{(\cdot)} - \hat{\theta}_{(-t)})^3}{6\left(\sum_{t=1}^{T}(\hat{\theta}_{(\cdot)} - \hat{\theta}_{(-t)})^2\right)^{3/2}}$$
where $\hat{\theta}_{(-t)}$ is the parameter estimate obtained by omitting the t-th observation and $\hat{\theta}_{(\cdot)} = \frac{1}{T}\sum_{i=1}^{T}\hat{\theta}_{(-t)}$.

5. **Calculate Corrected Percentiles:** Using the CDF of the standard normal $\Phi(\cdot)$, the corrected percentiles take the form:

$$\alpha_1 = \Phi\left(\Phi^{-1}(\hat{b}) + \frac{\Phi^{-1}(\hat{b}) + z_{\alpha/2}}{1 - \hat{a}(\Phi^{-1}(\hat{b}) + z_{\alpha/2})}\right)$$
$$\alpha_2 = \Phi\left(\Phi^{-1}(\hat{b}) + \frac{\Phi^{-1}(\hat{b}) + z_{1-\alpha/2}}{1 - \hat{a}(\Phi^{-1}(\hat{b}) + z_{1-\alpha/2})}\right)$$

where $z_{\alpha/2} = \Phi^{-1}(\alpha/2)$ and $z_{1-\alpha/2} = \Phi^{-1}(1-\alpha/2)$.

6. **Calculate BCa Confidence Interval:** The following BCa confidence interval accounts for both skewness and bias:

$$\left(\hat{\theta}^*_{(\alpha_1)}, \hat{\theta}^*_{(\alpha_2)}\right)$$

where $\hat{\theta}^*_{(\alpha_1)}$ and $\hat{\theta}^*_{(\alpha_2)}$ are the α_1 and α_2 percentiles of the bootstrap distribution of $\hat{\theta}^*$.

Other Confidence Intervals

As a sanity check for the BCa confidence intervals, we have included below p-values for the studentized t bootstrap (see np.boot() function documentation in nptest package [25]), a permutation test for the ratio of medians (function adapted from pairwise_comparison() in scoringutils package [26]), the distribution-free Bonett-Price CI for ratio of medians [27], Mood's median test [28] and a Wilcoxon signed-rank test (see wilcox.test(... , paired = TRUE) in stats package [29]). For R-code implemented here see ./scripts/getpvalues_func.R. Note that the main reasoning for not choosing a studentized t bootstrap was computational intensity. Permutation tests and the Wilcoxon signed-rank test were not chosen due to concerns over varying degrees of skewness in the absolute forecast errors produced by different models (see Figure B). The Mood's median test is only available for unpaired groups while here we want to make a test paired by target date. Bonett-Price ratio of medians CI is a reasonable choice if a closed-form solution is desired.

Figure B – **Histogram of Absolute Forecast Errors – Auvergne-Rhône-Alpes – 4 weeks horizon.** `COVIDici` and `ETS+ARIMA` have heavier tails than `AR−Lasso` and the Naive model which violate base assumptions for a permuation test of the ratio of two medians and Wilcoxon signed-rank test.

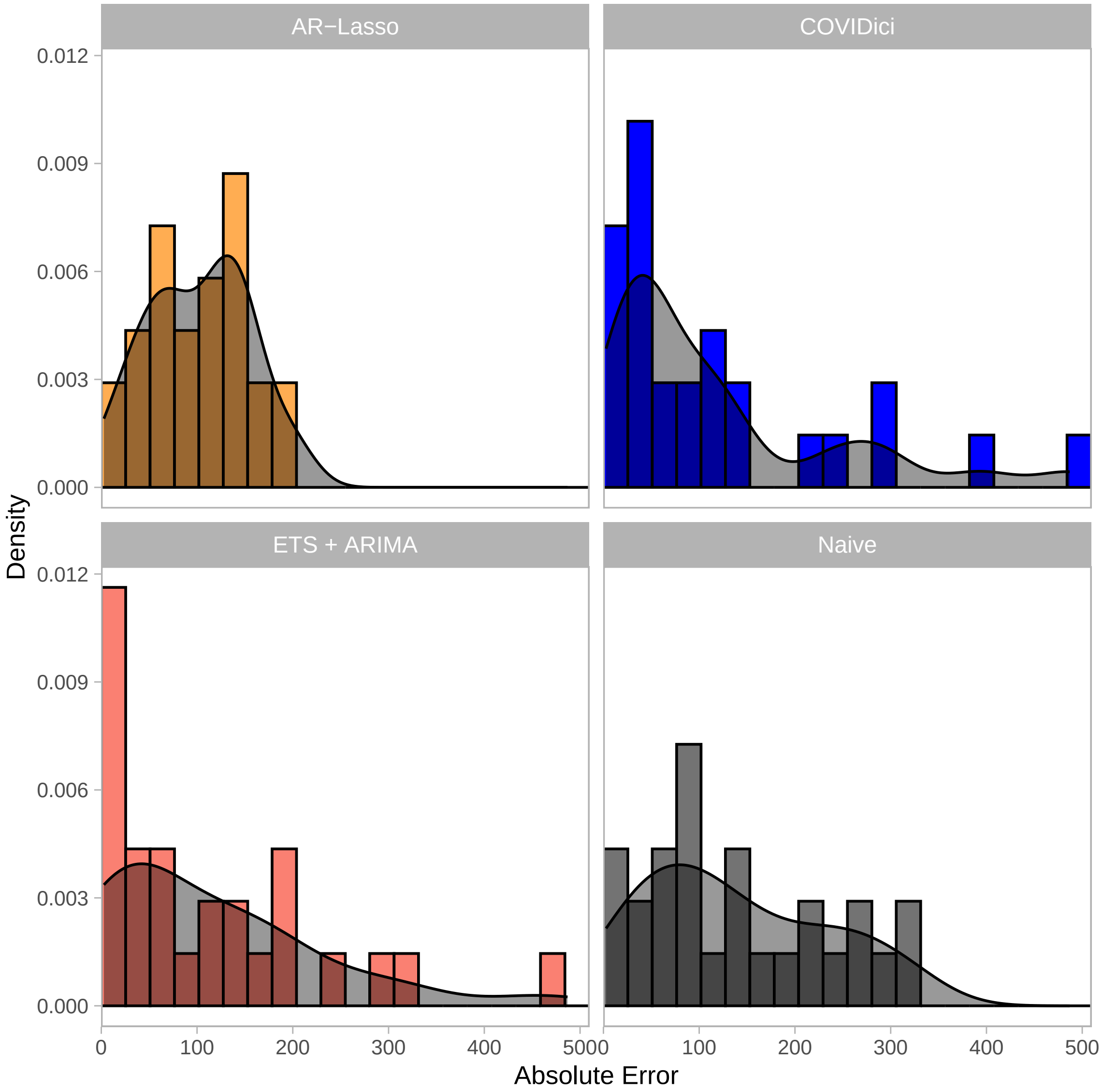

Table F – **Ratio of median AE (2 weeks ahead)**
Comparison of alternative hypothesis tests.

Region	Numerator	Denominator	n	Ratio	p-values					
					BCa	student	permute	Bonett-Price	Moods	Wilcoxon
Metropolitan France	AR-Lasso	Naive	38	0.50	< 0.001	< 0.001	0.00	0.00	0.01	< 0.001
	COVIDici	Naive	38	0.49	0.04	0.03	0.03	0.04	0.07	0.05
	COVIDici	AR-Lasso	38	0.97	0.95	0.96	0.95	0.93	0.36	0.18
	COVIDici	ETS + ARIMA	38	1.83	0.06	0.14	0.03	0.05	0.07	0.00
	ETS + ARIMA	Naive	38	0.27	< 0.001	< 0.001	0.00	< 0.001	< 0.001	< 0.001
	ETS + ARIMA	AR-Lasso	38	0.53	0.12	0.05	0.07	0.05	0.07	0.03
Auvergne-Rhône-Alpes	AR-Lasso	Naive	38	0.66	0.02	0.01	0.00	0.02	0.14	< 0.001
	COVIDici	Naive	38	0.71	0.19	0.11	0.19	0.17	0.14	0.33
	COVIDici	AR-Lasso	38	1.07	0.56	0.49	0.74	0.75	0.33	0.13
	COVIDici	ETS + ARIMA	38	1.19	0.48	0.55	0.32	0.57	0.24	0.03
	ETS + ARIMA	Naive	38	0.60	0.03	0.03	0.04	0.08	0.03	0.00
	ETS + ARIMA	AR-Lasso	38	0.90	0.68	0.85	0.44	0.72	0.24	0.53
Bourgogne-Franche-Comté	AR-Lasso	Naive	38	0.93	0.90	0.91	0.77	0.80	0.36	0.19
	COVIDici	Naive	38	1.15	0.44	0.36	0.49	0.68	0.33	0.74
	COVIDici	AR-Lasso	38	1.24	0.23	0.14	0.20	0.31	0.24	0.91
	COVIDici	ETS + ARIMA	38	1.47	0.07	0.53	0.06	0.16	0.14	0.12
	ETS + ARIMA	Naive	38	0.79	0.73	0.74	0.39	0.52	0.24	0.06
	ETS + ARIMA	AR-Lasso	38	0.85	0.81	0.81	0.53	0.57	0.36	0.14
Bretagne	AR-Lasso	Naive	38	0.62	0.03	0.00	0.01	0.00	0.01	0.00
	COVIDici	Naive	38	0.64	0.04	0.05	0.05	0.04	0.03	0.12
	COVIDici	AR-Lasso	38	1.04	0.93	0.98	0.88	0.86	0.36	0.89
	COVIDici	ETS + ARIMA	38	0.88	0.63	0.57	0.58	0.56	0.33	0.97
	ETS + ARIMA	Naive	38	0.73	0.07	0.02	0.05	0.05	0.07	0.01
	ETS + ARIMA	AR-Lasso	38	1.18	0.55	0.59	0.21	0.38	0.24	0.64
Centre-Val de Loire	AR-Lasso	Naive	38	0.78	0.36	0.37	0.23	0.30	0.33	0.00
	COVIDici	Naive	38	1.21	0.40	0.38	0.78	0.56	0.33	0.55
	COVIDici	AR-Lasso	38	1.55	0.16	0.24	0.34	0.19	0.33	0.14
	COVIDici	ETS + ARIMA	38	1.65	0.24	0.27	0.22	0.13	0.14	0.03
	ETS + ARIMA	Naive	38	0.73	0.48	0.48	0.17	0.23	0.14	< 0.001
	ETS + ARIMA	AR-Lasso	38	0.94	0.95	0.91	0.73	0.84	0.36	0.35
Corse	AR-Lasso	Naive	38	0.98	0.86	0.85	0.99	0.93	0.36	0.70
	COVIDici	Naive	38	1.34	0.21	0.21	0.15	0.34	0.24	0.10
	COVIDici	AR-Lasso	38	1.37	0.24	0.22	0.25	0.38	0.14	0.03
	COVIDici	ETS + ARIMA	38	1.56	0.11	0.05	0.02	0.12	0.14	< 0.001
	ETS + ARIMA	Naive	38	0.86	0.15	0.14	0.14	0.39	0.33	0.69
	ETS + ARIMA	AR-Lasso	38	0.88	0.42	0.47	0.58	0.67	0.33	0.68
Grand Est	AR-Lasso	Naive	38	0.71	0.13	0.12	0.03	0.11	0.14	< 0.001
	COVIDici	Naive	38	0.94	0.84	0.87	0.87	0.85	0.36	0.54
	COVIDici	AR-Lasso	38	1.33	0.19	0.33	0.11	0.20	0.14	0.11
	COVIDici	ETS + ARIMA	38	1.38	0.07	0.10	0.08	0.20	0.07	0.03
	ETS + ARIMA	Naive	38	0.68	0.08	0.12	0.08	0.23	0.07	0.00
	ETS + ARIMA	AR-Lasso	38	0.96	0.77	0.72	0.78	0.85	0.36	0.41
Hauts-de-France	AR-Lasso	Naive	38	0.49	0.00	0.00	0.00	0.00	0.03	< 0.001
	COVIDici	Naive	38	0.57	0.01	0.02	0.01	0.03	0.07	0.01
	COVIDici	AR-Lasso	38	1.15	0.44	0.38	0.64	0.50	0.33	0.46
	COVIDici	ETS + ARIMA	38	1.00	0.92	0.92	1.00	1.00	0.36	0.54
	ETS + ARIMA	Naive	38	0.57	0.03	0.05	0.01	0.05	0.14	< 0.001
	ETS + ARIMA	AR-Lasso	38	1.15	0.33	0.37	0.54	0.52	0.33	0.29
Normandie	AR-Lasso	Naive	38	0.83	0.57	0.45	0.36	0.38	0.33	0.02
	COVIDici	Naive	38	0.73	0.23	0.21	0.34	0.30	0.33	0.71
	COVIDici	AR-Lasso	38	0.87	0.33	0.29	0.79	0.61	0.33	0.21
	COVIDici	ETS + ARIMA	38	0.82	0.26	0.16	0.77	0.54	0.33	0.34
	ETS + ARIMA	Naive	38	0.89	0.92	0.98	0.74	0.75	0.36	0.13
	ETS + ARIMA	AR-Lasso	38	1.07	0.61	0.57	0.84	0.84	0.33	0.94
Nouvelle-Aquitaine	AR-Lasso	Naive	38	0.70	0.13	0.08	0.01	0.07	0.07	< 0.001
	COVIDici	Naive	38	1.00	0.86	0.85	1.00	0.99	0.36	0.61
	COVIDici	AR-Lasso	38	1.44	0.20	0.36	0.16	0.22	0.07	0.01
	COVIDici	ETS + ARIMA	38	1.35	0.23	0.48	0.06	0.28	0.14	0.00
	ETS + ARIMA	Naive	38	0.74	0.18	0.23	0.06	0.19	0.01	< 0.001
	ETS + ARIMA	AR-Lasso	38	1.06	0.69	0.63	0.76	0.82	0.33	0.34
Occitanie	AR-Lasso	Naive	38	0.43	< 0.001	< 0.001	0.00	< 0.001	0.01	< 0.001
	COVIDici	Naive	38	0.65	0.36	0.25	0.24	0.22	0.14	0.69
	COVIDici	AR-Lasso	38	1.52	0.19	0.41	0.15	0.22	0.14	0.03
	COVIDici	ETS + ARIMA	38	1.16	0.49	0.60	0.77	0.54	0.36	0.03
	ETS + ARIMA	Naive	38	0.56	0.01	0.01	0.01	0.02	0.01	0.01
	ETS + ARIMA	AR-Lasso	38	1.31	0.28	0.30	0.19	0.26	0.14	0.11
Pays de la Loire	AR-Lasso	Naive	38	0.83	0.13	0.05	0.04	0.11	0.14	0.13
	COVIDici	Naive	38	0.76	0.30	0.12	0.16	0.17	0.14	0.38
	COVIDici	AR-Lasso	38	0.92	0.69	0.63	0.73	0.71	0.33	0.92
	COVIDici	ETS + ARIMA	38	1.25	0.37	0.35	0.45	0.35	0.33	0.40
	ETS + ARIMA	Naive	38	0.61	0.01	0.00	0.03	0.00	0.07	0.04
	ETS + ARIMA	AR-Lasso	38	0.74	0.03	0.01	0.09	0.05	0.24	0.21
Provence-Alpes-Côte d'Azur	AR-Lasso	Naive	38	0.74	0.10	0.11	0.04	0.14	0.24	< 0.001
	COVIDici	Naive	38	1.03	0.83	0.82	0.98	0.93	0.36	0.97
	COVIDici	AR-Lasso	38	1.40	0.20	0.10	0.16	0.26	0.24	0.01
	COVIDici	ETS + ARIMA	38	2.41	0.01	0.01	0.00	0.01	0.24	< 0.001
	ETS + ARIMA	Naive	38	0.43	0.01	0.00	0.00	< 0.001	0.07	< 0.001
	ETS + ARIMA	AR-Lasso	38	0.58	0.17	0.04	0.02	0.03	0.24	0.30
Île-de-France	AR-Lasso	Naive	38	0.54	0.01	0.03	0.00	0.06	0.00	< 0.001
	COVIDici	Naive	38	0.73	0.08	0.31	0.07	0.27	0.03	0.01
	COVIDici	AR-Lasso	38	1.35	0.15	0.15	0.24	0.22	0.24	0.31
	COVIDici	ETS + ARIMA	38	1.79	0.02	0.00	0.04	0.02	0.14	0.10
	ETS + ARIMA	Naive	38	0.41	< 0.001	0.00	0.00	0.00	< 0.001	< 0.001
	ETS + ARIMA	AR-Lasso	38	0.75	0.30	0.25	0.41	0.36	0.24	0.39

Table G – **Ratio of median AE (4 weeks ahead)**
Comparison of alternative hypothesis tests.

					p-values					
**Region**	**n**	**Numerator**	**Denominator**	**Ratio**	**BCa**	**student**	**permute**	**Bonett-Price**	**Moods**	**Wilcoxon**
Metropolitan France	31	AR-Lasso	Naive	0.69	0.03	0.01	0.05	0.09	0.18	< 0.001
		COVIDici	Naive	0.59	0.06	0.07	0.07	0.10	0.09	0.06
		COVIDici	AR-Lasso	0.86	0.73	0.77	0.55	0.69	0.39	0.68
		COVIDici	ETS + ARIMA	1.07	0.90	0.84	1.00	0.81	0.39	0.71
		ETS + ARIMA	Naive	0.55	0.16	0.12	0.10	0.12	0.03	0.08
		ETS + ARIMA	AR-Lasso	0.80	0.48	0.72	0.41	0.60	0.09	0.74
Auvergne-Rhône-Alpes	31	AR-Lasso	Naive	1.22	0.41	0.12	0.48	0.40	0.39	0.15
		COVIDici	Naive	0.57	0.17	0.10	0.16	0.13	0.30	0.54
		COVIDici	AR-Lasso	0.47	0.03	0.01	0.02	0.01	0.18	0.81
		COVIDici	ETS + ARIMA	0.62	0.01	0.01	0.19	0.10	0.39	0.95
		ETS + ARIMA	Naive	0.93	0.96	0.96	0.89	0.86	0.39	0.23
		ETS + ARIMA	AR-Lasso	0.76	0.36	0.33	0.37	0.45	0.39	0.53
Bourgogne-Franche-Comté	31	AR-Lasso	Naive	1.36	0.21	0.14	0.08	0.23	0.18	0.53
		COVIDici	Naive	0.86	0.62	0.57	0.37	0.72	0.30	0.39
		COVIDici	AR-Lasso	0.63	0.12	0.12	0.07	0.23	0.03	0.10
		COVIDici	ETS + ARIMA	0.80	0.42	0.26	0.29	0.56	0.30	0.85
		ETS + ARIMA	Naive	1.07	0.49	0.47	0.86	0.81	0.39	0.20
		ETS + ARIMA	AR-Lasso	0.79	0.40	0.59	0.20	0.47	0.18	0.07
Bretagne	29	AR-Lasso	Naive	0.92	0.75	0.91	0.68	0.78	0.31	0.40
		COVIDici	Naive	0.59	0.05	0.06	0.11	0.09	0.03	0.28
		COVIDici	AR-Lasso	0.64	0.14	0.07	0.15	0.10	0.18	0.14
		COVIDici	ETS + ARIMA	0.66	0.12	0.09	0.08	0.14	0.08	0.37
		ETS + ARIMA	Naive	0.89	0.18	0.48	0.66	0.47	0.40	0.30
		ETS + ARIMA	AR-Lasso	0.97	0.69	0.71	1.00	0.91	0.40	0.88
Centre-Val de Loire	31	AR-Lasso	Naive	1.11	0.26	0.59	0.51	0.69	0.30	0.78
		COVIDici	Naive	0.51	0.04	0.05	0.12	0.08	0.18	0.15
		COVIDici	AR-Lasso	0.46	0.05	0.03	0.09	0.06	0.09	0.22
		COVIDici	ETS + ARIMA	0.79	0.59	0.42	0.28	0.38	0.18	0.48
		ETS + ARIMA	Naive	0.65	0.08	0.04	0.27	0.11	0.39	0.09
		ETS + ARIMA	AR-Lasso	0.59	0.04	0.01	0.11	0.07	0.18	0.39
Corse	30	AR-Lasso	Naive	1.17	0.38	0.22	0.75	0.61	0.36	0.13
		COVIDici	Naive	1.29	0.24	0.30	0.32	0.44	0.36	0.75
		COVIDici	AR-Lasso	1.10	0.89	0.95	0.58	0.81	0.24	0.06
		COVIDici	ETS + ARIMA	1.28	0.38	0.36	0.18	0.32	0.24	0.03
		ETS + ARIMA	Naive	1.01	0.65	0.64	0.95	0.97	0.41	0.49
		ETS + ARIMA	AR-Lasso	0.86	0.45	0.45	0.70	0.67	0.36	0.19
Grand Est	31	AR-Lasso	Naive	1.41	0.37	0.01	0.04	0.09	0.30	0.06
		COVIDici	Naive	1.15	0.47	0.21	0.66	0.67	0.39	0.28
		COVIDici	AR-Lasso	0.81	0.35	0.34	0.17	0.49	0.18	0.62
		COVIDici	ETS + ARIMA	1.14	0.27	0.18	0.32	0.60	0.39	0.56
		ETS + ARIMA	Naive	1.01	0.83	0.82	0.91	0.98	0.39	0.10
		ETS + ARIMA	AR-Lasso	0.71	0.01	0.01	0.02	0.01	0.00	0.53
Hauts-de-France	30	AR-Lasso	Naive	1.23	0.30	0.23	0.72	0.40	0.36	0.17
		COVIDici	Naive	0.62	0.12	0.13	0.10	0.12	0.02	0.02
		COVIDici	AR-Lasso	0.51	0.04	0.02	0.03	0.01	0.05	0.06
		COVIDici	ETS + ARIMA	0.76	0.26	0.26	0.20	0.26	0.13	0.06
		ETS + ARIMA	Naive	0.82	0.71	0.59	0.51	0.55	0.36	0.19
		ETS + ARIMA	AR-Lasso	0.67	0.16	0.12	0.35	0.21	0.24	0.98
Normandie	31	AR-Lasso	Naive	0.95	0.55	0.58	0.85	0.83	0.39	0.43
		COVIDici	Naive	0.58	0.02	0.02	0.04	0.04	0.09	0.18
		COVIDici	AR-Lasso	0.61	0.05	0.02	0.03	0.01	0.09	0.12
		COVIDici	ETS + ARIMA	0.74	0.22	0.21	0.22	0.36	0.30	0.42
		ETS + ARIMA	Naive	0.78	0.49	0.42	0.50	0.52	0.30	0.19
		ETS + ARIMA	AR-Lasso	0.82	0.40	0.57	0.39	0.53	0.18	0.57
Nouvelle-Aquitaine	31	AR-Lasso	Naive	0.90	0.06	0.10	0.08	0.50	0.18	0.15
		COVIDici	Naive	0.79	0.46	0.31	0.36	0.51	0.30	0.69
		COVIDici	AR-Lasso	0.88	0.59	0.48	0.43	0.70	0.30	0.50
		COVIDici	ETS + ARIMA	1.08	0.83	0.89	0.62	0.77	0.30	0.26
		ETS + ARIMA	Naive	0.72	0.08	0.06	0.19	0.22	0.09	0.41
		ETS + ARIMA	AR-Lasso	0.81	0.29	0.28	0.26	0.34	0.09	0.50
Occitanie	31	AR-Lasso	Naive	0.68	0.02	0.00	0.01	0.01	0.03	< 0.001
		COVIDici	Naive	0.61	0.04	0.06	0.08	0.12	0.09	0.39
		COVIDici	AR-Lasso	0.89	0.56	0.49	0.45	0.71	0.39	0.38
		COVIDici	ETS + ARIMA	0.63	0.13	0.04	0.23	0.12	0.39	0.98
		ETS + ARIMA	Naive	0.96	0.88	0.94	0.73	0.88	0.30	0.37
		ETS + ARIMA	AR-Lasso	1.40	0.52	0.31	0.19	0.23	0.39	0.06
Pays de la Loire	31	AR-Lasso	Naive	1.04	0.73	0.62	0.80	0.86	0.39	0.39
		COVIDici	Naive	0.83	0.32	0.37	0.58	0.57	0.30	0.69
		COVIDici	AR-Lasso	0.80	0.15	0.13	0.45	0.45	0.30	0.78
		COVIDici	ETS + ARIMA	1.10	0.78	0.73	0.82	0.79	0.39	0.65
		ETS + ARIMA	Naive	0.76	0.10	0.11	0.26	0.21	0.39	0.22
		ETS + ARIMA	AR-Lasso	0.73	0.14	0.09	0.32	0.22	0.30	0.64
Provence-Alpes-Côte d'Azur	31	AR-Lasso	Naive	0.87	0.56	0.53	0.35	0.38	0.30	0.01
		COVIDici	Naive	0.65	0.61	0.43	0.43	0.32	0.30	0.90
		COVIDici	AR-Lasso	0.75	0.64	0.55	0.29	0.42	0.30	0.31
		COVIDici	ETS + ARIMA	0.84	0.69	0.68	0.66	0.62	0.39	0.71
		ETS + ARIMA	Naive	0.78	0.53	0.48	0.34	0.49	0.30	0.21
		ETS + ARIMA	AR-Lasso	0.89	0.58	0.70	0.57	0.73	0.39	0.95
Île-de-France	31	AR-Lasso	Naive	0.97	0.84	0.73	0.76	0.94	0.30	0.01
		COVIDici	Naive	0.81	0.58	0.84	0.48	0.57	0.18	0.05
		COVIDici	AR-Lasso	0.84	0.59	0.61	0.57	0.54	0.30	0.61
		COVIDici	ETS + ARIMA	1.05	0.73	0.65	1.00	0.86	0.39	0.46
		ETS + ARIMA	Naive	0.78	0.33	0.51	0.24	0.48	0.18	0.12
		ETS + ARIMA	AR-Lasso	0.80	0.19	0.20	0.39	0.36	0.30	0.75

Table H – **Ratio of median WIS (2 weeks ahead)**
Comparison of alternative hypothesis tests.

					p-values					
**Region**	**n**	**Numerator**	**Denominator**	**Ratio**	**BCa**	**student**	**permute**	**Bonett-Price**	**Moods**	**Wilcoxon**
Metropolitan France	38	AR-Lasso	Naive	0.48	0.01	0.01	0.00	0.01	0.07	< 0.001
		COVIDici	Naive	0.66	0.33	0.22	0.31	0.31	0.07	0.29
		COVIDici	AR-Lasso	1.36	0.44	0.51	0.44	0.48	0.33	0.03
		COVIDici	ETS + ARIMA	2.23	0.01	0.01	0.00	0.01	0.03	< 0.001
		ETS + ARIMA	Naive	0.29	< 0.001	< 0.001	0.00	< 0.001	< 0.001	< 0.001
		ETS + ARIMA	AR-Lasso	0.61	0.27	0.13	0.11	0.14	0.14	0.06
Auvergne-Rhône-Alpes	38	AR-Lasso	Naive	0.70	0.08	0.07	0.03	0.06	0.24	0.00
		COVIDici	Naive	0.98	0.72	0.74	0.96	0.94	0.36	0.87
		COVIDici	AR-Lasso	1.40	0.17	0.23	0.06	0.20	0.14	0.01
		COVIDici	ETS + ARIMA	1.20	0.45	0.65	0.26	0.52	0.14	0.00
		ETS + ARIMA	Naive	0.82	0.41	0.40	0.25	0.44	0.33	0.01
		ETS + ARIMA	AR-Lasso	1.17	0.34	0.36	0.58	0.46	0.33	0.79
Bourgogne-Franche-Comté	38	AR-Lasso	Naive	0.97	0.94	1.00	0.84	0.92	0.36	0.10
		COVIDici	Naive	1.60	0.20	0.17	0.12	0.21	0.14	0.54
		COVIDici	AR-Lasso	1.65	0.01	0.01	0.01	0.02	0.07	0.14
		COVIDici	ETS + ARIMA	1.30	0.23	0.13	0.38	0.34	0.24	0.54
		ETS + ARIMA	Naive	1.23	0.66	0.56	0.34	0.59	0.33	0.81
		ETS + ARIMA	AR-Lasso	1.27	0.48	0.61	0.24	0.46	0.24	0.24
Bretagne	38	AR-Lasso	Naive	0.55	0.03	< 0.001	0.01	< 0.001	0.01	< 0.001
		COVIDici	Naive	0.76	0.18	0.25	0.23	0.37	0.14	0.42
		COVIDici	AR-Lasso	1.38	0.31	0.16	0.12	0.19	0.14	0.13
		COVIDici	ETS + ARIMA	1.14	0.64	0.55	0.47	0.64	0.33	0.11
		ETS + ARIMA	Naive	0.66	0.06	0.00	0.03	0.02	0.03	< 0.001
		ETS + ARIMA	AR-Lasso	1.21	0.19	0.34	0.13	0.27	0.14	0.48
Centre-Val de Loire	38	AR-Lasso	Naive	0.77	0.51	0.49	0.24	0.34	0.33	< 0.001
		COVIDici	Naive	1.59	0.19	0.07	0.32	0.20	0.24	0.76
		COVIDici	AR-Lasso	2.07	0.05	0.11	0.06	0.04	0.14	0.03
		COVIDici	ETS + ARIMA	1.33	0.45	0.54	0.22	0.38	0.14	0.00
		ETS + ARIMA	Naive	1.20	0.40	0.16	0.57	0.52	0.33	0.00
		ETS + ARIMA	AR-Lasso	1.55	0.07	0.07	0.23	0.09	0.24	1.00
Corse	38	AR-Lasso	Naive	0.90	0.54	0.54	0.73	0.67	0.33	0.44
		COVIDici	Naive	2.24	0.00	0.01	0.00	0.01	0.07	< 0.001
		COVIDici	AR-Lasso	2.50	< 0.001	0.01	0.00	0.01	0.03	< 0.001
		COVIDici	ETS + ARIMA	1.73	0.01	0.03	0.00	0.09	0.24	< 0.001
		ETS + ARIMA	Naive	1.30	0.09	0.11	0.11	0.14	0.24	0.62
		ETS + ARIMA	AR-Lasso	1.45	0.05	0.09	0.12	0.08	0.14	0.08
Grand Est	38	AR-Lasso	Naive	0.62	0.04	0.06	0.01	0.09	0.03	< 0.001
		COVIDici	Naive	1.34	0.46	0.40	0.30	0.42	0.24	0.61
		COVIDici	AR-Lasso	2.15	0.01	0.01	0.01	< 0.001	0.00	0.01
		COVIDici	ETS + ARIMA	1.41	0.16	0.39	0.09	0.21	0.14	0.01
		ETS + ARIMA	Naive	0.95	0.99	0.98	0.84	0.90	0.36	0.02
		ETS + ARIMA	AR-Lasso	1.53	0.07	0.10	0.07	0.07	0.03	0.84
Hauts-de-France	38	AR-Lasso	Naive	0.41	0.00	0.00	0.01	0.00	0.07	< 0.001
		COVIDici	Naive	0.75	0.30	0.34	0.27	0.38	0.33	0.08
		COVIDici	AR-Lasso	1.82	0.02	0.00	0.02	0.00	0.14	0.52
		COVIDici	ETS + ARIMA	1.24	0.41	0.36	0.43	0.25	0.33	0.06
		ETS + ARIMA	Naive	0.61	0.12	0.10	0.02	0.09	0.14	< 0.001
		ETS + ARIMA	AR-Lasso	1.47	0.03	0.01	0.20	0.02	0.24	0.74
Normandie	38	AR-Lasso	Naive	0.69	0.17	0.17	0.15	0.15	0.33	0.01
		COVIDici	Naive	1.04	0.97	1.00	0.99	0.93	0.36	0.55
		COVIDici	AR-Lasso	1.51	0.11	0.10	0.12	0.18	0.14	0.01
		COVIDici	ETS + ARIMA	0.90	0.46	0.43	0.69	0.69	0.33	0.61
		ETS + ARIMA	Naive	1.15	0.77	0.79	0.62	0.61	0.36	0.81
		ETS + ARIMA	AR-Lasso	1.68	0.01	0.02	0.01	0.01	0.07	< 0.001
Nouvelle-Aquitaine	38	AR-Lasso	Naive	0.62	0.07	0.03	0.00	0.04	0.03	< 0.001
		COVIDici	Naive	1.53	0.15	0.19	0.38	0.19	0.24	0.13
		COVIDici	AR-Lasso	2.48	0.02	0.06	0.00	0.00	0.03	< 0.001
		COVIDici	ETS + ARIMA	1.70	0.02	0.05	0.00	0.01	0.14	< 0.001
		ETS + ARIMA	Naive	0.90	0.56	0.71	0.67	0.69	0.33	0.00
		ETS + ARIMA	AR-Lasso	1.46	0.24	0.18	0.06	0.09	0.14	0.08
Occitanie	38	AR-Lasso	Naive	0.32	< 0.001	< 0.001	0.00	< 0.001	0.00	< 0.001
		COVIDici	Naive	0.72	0.48	0.39	0.38	0.41	0.24	0.83
		COVIDici	AR-Lasso	2.27	0.01	0.28	0.02	0.02	0.01	0.00
		COVIDici	ETS + ARIMA	1.38	0.16	0.41	0.06	0.21	0.14	< 0.001
		ETS + ARIMA	Naive	0.52	0.02	0.00	0.01	0.01	0.01	< 0.001
		ETS + ARIMA	AR-Lasso	1.64	0.01	0.01	0.01	0.01	0.03	0.10
Pays de la Loire	38	AR-Lasso	Naive	0.74	0.07	0.02	0.02	0.02	0.07	0.02
		COVIDici	Naive	0.92	0.82	0.77	0.71	0.76	0.33	0.98
		COVIDici	AR-Lasso	1.24	0.45	0.57	0.35	0.41	0.24	0.16
		COVIDici	ETS + ARIMA	1.02	0.94	0.93	0.95	0.95	0.36	0.21
		ETS + ARIMA	Naive	0.90	0.54	0.56	0.37	0.56	0.24	0.05
		ETS + ARIMA	AR-Lasso	1.22	0.06	0.04	0.16	0.11	0.24	0.67
Provence-Alpes-Côte d'Azur	38	AR-Lasso	Naive	0.65	0.07	0.09	0.01	0.12	0.07	< 0.001
		COVIDici	Naive	1.36	0.39	0.32	0.52	0.52	0.33	0.47
		COVIDici	AR-Lasso	2.10	0.02	0.03	0.00	0.04	0.07	< 0.001
		COVIDici	ETS + ARIMA	1.37	0.30	0.23	0.08	0.34	0.24	< 0.001
		ETS + ARIMA	Naive	1.00	0.83	0.83	0.98	1.00	0.36	0.03
		ETS + ARIMA	AR-Lasso	1.54	0.00	0.02	0.01	0.00	0.24	0.02
Île-de-France	38	AR-Lasso	Naive	0.52	0.04	0.04	0.01	0.06	0.03	< 0.001
		COVIDici	Naive	1.02	0.73	0.70	0.88	0.95	0.33	0.13
		COVIDici	AR-Lasso	1.95	0.01	0.00	0.04	0.01	0.07	0.01
		COVIDici	ETS + ARIMA	1.95	0.01	0.00	0.00	0.01	0.07	0.01
		ETS + ARIMA	Naive	0.53	0.00	0.03	0.00	0.03	0.00	< 0.001
		ETS + ARIMA	AR-Lasso	1.00	0.98	1.00	0.98	0.99	0.36	0.58

Table I – **Ratio of median WIS (4 weeks ahead)**
Comparison of alternative hypothesis tests.

					p-values					
**Region**	**n**	**Numerator**	**Denominator**	**Ratio**	**BCa**	**student**	**permute**	**Bonett-Price**	**Moods**	**Wilcoxon**
Metropolitan France	31	AR-Lasso	Naive	0.70	0.14	0.09	0.08	0.10	0.30	0.00
		COVIDici	Naive	0.70	0.28	0.27	0.24	0.27	0.18	0.32
		COVIDici	AR-Lasso	1.01	1.00	0.95	1.00	0.97	0.39	0.65
		COVIDici	ETS + ARIMA	1.43	0.17	0.14	0.13	0.11	0.18	0.01
		ETS + ARIMA	Naive	0.49	0.08	0.03	0.05	0.05	0.03	0.01
		ETS + ARIMA	AR-Lasso	0.71	0.45	0.41	0.43	0.42	0.30	0.25
Auvergne-Rhône-Alpes	31	AR-Lasso	Naive	1.29	0.42	0.17	0.50	0.42	0.39	0.13
		COVIDici	Naive	0.87	0.66	0.71	0.91	0.74	0.39	0.74
		COVIDici	AR-Lasso	0.68	0.40	0.14	0.40	0.19	0.39	0.32
		COVIDici	ETS + ARIMA	0.89	0.27	0.36	1.00	0.66	0.39	0.01
		ETS + ARIMA	Naive	0.97	0.92	0.88	1.00	0.95	0.39	0.10
		ETS + ARIMA	AR-Lasso	0.75	0.41	0.42	0.48	0.40	0.30	0.31
Bourgogne-Franche-Comté	31	AR-Lasso	Naive	1.55	0.13	0.13	0.06	0.19	0.09	0.93
		COVIDici	Naive	1.27	0.52	0.42	0.54	0.60	0.30	0.95
		COVIDici	AR-Lasso	0.82	0.64	0.67	0.44	0.65	0.39	0.62
		COVIDici	ETS + ARIMA	1.09	0.77	0.51	0.83	0.83	0.39	0.61
		ETS + ARIMA	Naive	1.17	0.90	0.73	0.35	0.63	0.30	0.68
		ETS + ARIMA	AR-Lasso	0.75	0.26	0.32	0.48	0.44	0.30	0.93
Bretagne	29	AR-Lasso	Naive	0.86	0.81	0.80	0.65	0.66	0.40	0.14
		COVIDici	Naive	0.81	0.60	0.62	0.50	0.55	0.31	0.72
		COVIDici	AR-Lasso	0.95	0.88	0.86	0.87	0.86	0.40	0.98
		COVIDici	ETS + ARIMA	0.79	0.22	0.20	0.56	0.35	0.40	0.43
		ETS + ARIMA	Naive	1.03	0.47	0.38	1.00	0.88	0.40	0.02
		ETS + ARIMA	AR-Lasso	1.20	0.36	0.43	0.89	0.52	0.40	0.83
Centre-Val de Loire	31	AR-Lasso	Naive	1.05	0.79	0.77	1.00	0.86	0.39	0.14
		COVIDici	Naive	0.61	0.28	0.25	0.39	0.28	0.39	0.47
		COVIDici	AR-Lasso	0.58	0.23	0.16	0.34	0.23	0.30	0.99
		COVIDici	ETS + ARIMA	1.03	0.89	0.82	1.00	0.93	0.39	0.25
		ETS + ARIMA	Naive	0.60	0.05	0.07	0.21	0.08	0.30	0.01
		ETS + ARIMA	AR-Lasso	0.56	0.04	0.01	0.16	0.04	0.30	0.16
Corse	30	AR-Lasso	Naive	1.03	0.73	0.71	0.94	0.94	0.41	0.06
		COVIDici	Naive	1.81	0.03	0.01	0.06	0.05	0.24	0.02
		COVIDici	AR-Lasso	1.76	0.07	0.10	0.01	0.10	0.05	0.00
		COVIDici	ETS + ARIMA	1.71	0.02	0.02	0.04	0.04	0.24	0.03
		ETS + ARIMA	Naive	1.06	0.74	0.71	0.83	0.85	0.36	0.30
		ETS + ARIMA	AR-Lasso	1.03	0.86	0.85	0.86	0.93	0.41	0.09
Grand Est	31	AR-Lasso	Naive	1.62	0.14	0.00	0.04	0.11	0.39	0.03
		COVIDici	Naive	1.75	0.27	0.04	0.30	0.14	0.30	0.84
		COVIDici	AR-Lasso	1.08	0.85	0.88	0.60	0.82	0.30	0.53
		COVIDici	ETS + ARIMA	1.57	0.16	0.02	0.08	0.11	0.18	0.30
		ETS + ARIMA	Naive	1.11	0.58	0.42	0.77	0.77	0.39	0.06
		ETS + ARIMA	AR-Lasso	0.69	0.06	0.05	0.12	0.17	0.03	0.47
Hauts-de-France	30	AR-Lasso	Naive	1.09	0.55	0.54	0.95	0.81	0.41	0.12
		COVIDici	Naive	0.91	0.78	0.83	0.62	0.78	0.24	0.16
		COVIDici	AR-Lasso	0.83	0.48	0.55	0.53	0.56	0.24	0.63
		COVIDici	ETS + ARIMA	1.09	0.65	0.54	0.63	0.76	0.41	0.63
		ETS + ARIMA	Naive	0.83	0.71	0.73	0.62	0.65	0.24	0.04
		ETS + ARIMA	AR-Lasso	0.77	0.40	0.41	0.56	0.50	0.24	0.95
Normandie	31	AR-Lasso	Naive	0.77	0.28	0.22	0.60	0.38	0.30	0.13
		COVIDici	Naive	0.74	0.25	0.30	0.50	0.44	0.30	0.57
		COVIDici	AR-Lasso	0.96	0.75	0.75	0.82	0.89	0.39	0.90
		COVIDici	ETS + ARIMA	0.50	< 0.001	< 0.001	0.00	0.01	0.03	0.00
		ETS + ARIMA	Naive	1.48	0.18	0.14	0.17	0.23	0.30	0.06
		ETS + ARIMA	AR-Lasso	1.91	0.00	0.00	0.00	0.00	0.18	0.00
Nouvelle-Aquitaine	31	AR-Lasso	Naive	0.71	0.04	0.00	0.01	0.04	0.09	0.00
		COVIDici	Naive	0.97	0.87	0.78	1.00	0.94	0.39	0.53
		COVIDici	AR-Lasso	1.36	0.29	0.17	0.22	0.42	0.18	0.03
		COVIDici	ETS + ARIMA	1.46	0.13	0.05	0.09	0.20	0.18	0.00
		ETS + ARIMA	Naive	0.66	0.05	0.02	0.08	0.08	0.09	0.07
		ETS + ARIMA	AR-Lasso	0.93	0.57	0.54	0.67	0.73	0.30	0.50
Occitanie	31	AR-Lasso	Naive	0.50	< 0.001	< 0.001	0.00	< 0.001	0.01	< 0.001
		COVIDici	Naive	0.65	0.21	0.14	0.20	0.20	0.18	0.64
		COVIDici	AR-Lasso	1.31	0.34	0.25	0.51	0.42	0.30	0.09
		COVIDici	ETS + ARIMA	1.10	0.76	0.64	1.00	0.74	0.39	0.05
		ETS + ARIMA	Naive	0.59	0.06	0.02	0.06	0.02	0.09	0.07
		ETS + ARIMA	AR-Lasso	1.19	0.34	0.52	0.58	0.52	0.39	0.11
Pays de la Loire	31	AR-Lasso	Naive	1.02	0.68	0.66	0.86	0.94	0.39	0.13
		COVIDici	Naive	1.18	0.89	0.66	0.49	0.70	0.39	0.76
		COVIDici	AR-Lasso	1.15	0.51	0.84	0.36	0.69	0.18	0.20
		COVIDici	ETS + ARIMA	1.03	0.89	0.85	0.83	0.93	0.39	0.46
		ETS + ARIMA	Naive	1.14	0.29	0.41	0.87	0.60	0.39	0.74
		ETS + ARIMA	AR-Lasso	1.12	0.79	0.67	0.54	0.65	0.39	0.43
Provence-Alpes-Côte d'Azur	31	AR-Lasso	Naive	0.66	0.07	0.05	0.01	0.04	0.18	< 0.001
		COVIDici	Naive	0.78	0.79	0.72	0.70	0.60	0.30	0.66
		COVIDici	AR-Lasso	1.19	0.74	0.70	0.63	0.64	0.39	0.02
		COVIDici	ETS + ARIMA	0.88	0.78	0.79	0.44	0.65	0.39	0.02
		ETS + ARIMA	Naive	0.88	0.82	0.73	0.82	0.69	0.39	0.11
		ETS + ARIMA	AR-Lasso	1.35	0.20	0.17	0.17	0.13	0.09	0.15
Île-de-France	31	AR-Lasso	Naive	1.05	0.62	0.53	1.00	0.91	0.39	0.01
		COVIDici	Naive	1.12	0.64	0.49	1.00	0.80	0.39	0.21
		COVIDici	AR-Lasso	1.07	0.84	0.93	0.90	0.85	0.39	0.39
		COVIDici	ETS + ARIMA	1.48	0.06	0.09	0.11	0.15	0.30	0.26
		ETS + ARIMA	Naive	0.76	0.47	0.55	0.32	0.56	0.18	0.03
		ETS + ARIMA	AR-Lasso	0.72	0.23	0.10	0.34	0.23	0.18	0.88

References

[1] Sofonea MT, Reyné B, Elie B, Djidjou-Demasse R, Selinger C, Michalakis Y, et al. Memory is key in capturing COVID-19 epidemiological dynamics. Epidemics. 2021; p. 100459. doi:10.1016/j.epidem.2021.100459.

[2] INSEE. Estimation de la population au 1er janvier 2020 | INSEE; 2020. Available from: https://www.insee.fr/fr/statistiques/1893198.

[3] Haim-Boukobza S, Roquebert B, Trombert-Paolantoni S, Lecorche E, Verdurme L, Foulongne V, et al. Detecting Rapid Spread of SARS-CoV-2 Variants, France, January 26-February 16, 2021. Emerging Infectious Diseases. 2021;27(5):10.

[4] Roquebert B, Haim-Boukobza S, Trombert-Paolantoni S, Lecorche E, Verdurme L, Foulonge V, et al. SARS-CoV-2 variants of concern are associated with lower RT-PCR amplification cycles between January and March 2021 in France. medRxiv. 2021; p. 2021.03.19.21253971. doi:10.1101/2021.03.19.21253971.

[5] Alizon S, Haim-Boukobza S, Foulongne V, Verdurme L, Trombert-Paolantoni S, Lecorche E, et al. Rapid spread of the SARS-CoV-2 Delta variant in some French regions, June 2021. Eurosurveillance. 2021;26(28):2100573. doi:10.2807/1560-7917.ES.2021.26.28.2100573.

[6] Verity R, Okell LC, Dorigatti I, Winskill P, Whittaker C, Imai N, et al. Estimates of the severity of coronavirus disease 2019: a model-based analysis. The Lancet Infectious Diseases. 2020; p. S1473309920302437. doi:10.1016/S1473-3099(20)30243-7.

[7] Santé Publique France. COVID-19 : Point épidémiologique hebdomadaire du 7 mai 2020. santepubliquefrance.fr; 2020. Available from: https://www.santepubliquefrance.fr/content/download/250807/2596023.

[8] Salje H, Kiem CT, Lefrancq N, Courtejoie N, Bosetti P, Paireau J, et al. Estimating the burden of SARS-CoV-2 in France. Science. 2020;doi:10.1126/science.abc3517.

[9] Santé publique France. Données relatives aux personnes vaccinées contre la Covid-19 (VAC-SI) - data.gouv.fr; 2021. Available from: https://www.data.gouv.fr/fr/datasets/donnees-relatives-aux-personnes-vaccinees-contre-la-covid-19-1/.

[10] Dagan N, Barda N, Kepten E, Miron O, Perchik S, Katz MA, et al. BNT162b2 mRNA Covid-19 Vaccine in a Nationwide Mass Vaccination Setting. New England Journal of Medicine. 2021;doi:10.1056/NEJMoa2101765.

[11] Santé publique France. Données hospitalières relatives à l'épidémie de COVID-19 (SI-VIC) - data.gouv.fr; 2020. Available from: https://www.data.gouv.fr/fr/datasets/donnees-hospitalieres-relatives-a-lepidemie-de-covid-19/.

[12] Nishiura H, Linton NM, Akhmetzhanov AR. Serial interval of novel coronavirus (COVID-19) infections. International Journal of Infectious Diseases. 2020;93:284–286. doi:10.1016/j.ijid.2020.02.060.

[13] Cori A, Valleron AJ, Carrat F, Scalia Tomba G, Thomas G, Boëlle PY. Estimating influenza latency and infectious period durations using viral excretion data. Epidemics. 2012;4(3):132–138. doi:10.1016/j.epidem.2012.06.001.

[14] Thompson RN, Stockwin JE, van Gaalen RD, Polonsky JA, Kamvar ZN, Demarsh PA, et al. Improved inference of time-varying reproduction numbers during infectious disease outbreaks. Epidemics. 2019;29:100356. doi:10.1016/j.epidem.2019.100356.

[15] Hartig F, Minunno F, Paul S. BayesianTools: General-Purpose MCMC and SMC Samplers and Tools for Bayesian Statistics; 2020. Available from: https://CRAN.R-project.org/package=BayesianTools.

[16] Cramer EY, Huang Y, Wang Y, Ray EL, Cornell M, Bracher J, et al. The United States COVID-19 Forecast Hub dataset. medRxiv. 2021;doi:10.1101/2021.11.04.21265886.

[17] Santé Publique France. Website: Synthese des indicateurs de suivi de lepidemie Covid-19; 2023. Available from: https://static.data.gouv.fr/

resources/synthese-des-indicateurs-de-suivi-de-lepidemie-covid-19/20230630-155909/table-indicateurs-open-data-dep-2023-06-30-17h59.csv.

[18] Santé Publique France. Website: Données relatives aux personnes vaccinées contre la Covid-19; 2023. Available from: https://static.data.gouv.fr/resources/donnees-relatives-aux-personnes-vaccinees-contre-la-covid-19-1/20230713-135210/vacsi-s-a-dep-2023-07-13-15h51.csv.

[19] Santé Publique France. Website: Données relatives aux personnes vaccinées contre la Covid-19; 2023. Available from: https://static.data.gouv.fr/resources/donnees-relatives-aux-personnes-vaccinees-contre-la-covid-19-1/20230713-135120/vacsi-s-a-reg-2023-07-13-15h51.csv.

[20] Santé Publique France. Website: Données relatives aux personnes vaccinées contre la Covid-19; 2023. Available from: https://static.data.gouv.fr/resources/donnees-relatives-aux-personnes-vaccinees-contre-la-covid-19-1/20230713-135109/vacsi-s-a-fra-2023-07-13-15h51.csv.

[21] Massey A, Boennec C, Restrepo-Ortiz C, Blanchet C, Alizon S, Sofonea MT. COVIDici: Real-time forecasting of COVID-19-related hospital strain in France using a non-Markovian mechanistic model; 2023. Available from: https://doi.org/10.5281/zenodo.7641132.

[22] Hyndman RJ, Athanasopoulos G. Forecasting: principles and practice, 3rd edition. Melbourne, Australia: bookdown; 2021. Available from: https://OTexts.com/fpp3.

[23] Bracher J, Ray EL, Gneiting T, Reich NG. Evaluating epidemic forecasts in an interval format. PLOS Computational Biology. 2021;17(2):e1008618. doi:10.1371/journal.pcbi.1008618.

[24] Jordan A, Krüger F, Lerch S. Evaluating Probabilistic Forecasts with scoringRules. Journal of Statistical Software. 2019;90(12):1–37. doi:10.18637/jss.v090.i12.

[25] Helwig NE. nptest: Nonparametric Bootstrap and Permutation Tests; 2023. Available from: https://CRAN.R-project.org/package=nptest.

[26] Bosse NI, Gruson H, Cori A, van Leeuwen E, Funk S, Abbott S. Evaluating Forecasts with scoringutils in R. arXiv. 2022;doi:10.48550/ARXIV.2205.07090.

[27] Bonett DG, Price RM. Confidence Intervals for Ratios of Means and Medians. Journal of Educational and Behavioral Statistics. 2020;45:750–770. doi:10.3102/1076998620934125.

[28] Sweet DL. nonpar: A Collection of Nonparametric Hypothesis Tests; 2020. Available from: https://CRAN.R-project.org/package=nonpar.

[29] R Core Team. R: A Language and Environment for Statistical Computing; 2023. Available from: https://www.R-project.org/.

List of Figures

A **Artistic representation of the survivor bias that may occur after a restrictive governmental intervention.** 14

B **Histogram of Absolute Forecast Errors – Auvergne-Rhône-Alpes – 4 weeks horizon.** `COVIDici` and `ETS+ARIMA` have heavier tails than `AR-Lasso` and the Naive model which violate base assumptions for a permuation test of the ratio of two medians and Wilcoxon signed-rank test. 18

List of Tables

A **Main clinical-epidemiological compartments/densities simulated by the `COVIDici` model.** (NB: these quantities vary across calendar time t, scales and location ℓ – which are implicit here for the sake of readability). 2

B **Key parameters inferred by the `COVIDici` model.** The national priors were provided by the fitting/inference procedure of the $\texttt{COVIDSIM}-\texttt{FR}$ model, according to [1]. (NB: these quantities vary across calendar time t, scales and location ℓ – which are implicit here for the sake of readability). 3

C **Main constrained quantities of the `COVIDici` model.** 4

D **Main distributions involved in the `COVIDici` model.** 5

E **Additional notation table.** 5

F **Ratio of median AE (2 weeks ahead)** Comparison of alternative hypothesis tests. 19

G **Ratio of median AE (4 weeks ahead)** Comparison of alternative hypothesis tests. 20

H **Ratio of median WIS (2 weeks ahead)** Comparison of alternative hypothesis tests. 21

I **Ratio of median WIS (4 weeks ahead)** Comparison of alternative hypothesis tests. 22
