# Supplementary material for: Real-time forecasting of COVID-19-related hospital strain in France using a non-Markovian mechanistic model
Source: PLoS Comput Biol. 2024 May 17;20(5):e1012124. doi: 10.1371/journal.pcbi.1012124 (PMC11139328; doi:10.1371/journal.pcbi.1012124)
Supplement: Attachment — Submitted filename: Response_to_reviewersPLOScompbiol.pdf [file pcbi.1012124.s002.pdf]

Manuscript ID: PCOMPBIOL-D-23-01056

Real-time forecasting of COVID-19-related hospital strain in France using a non-Markovian mechanistic model

by Alexander Massey, Corentin Boennec, Claudia Ximena Restrepo-Ortiz, Christophe Blanchet, Samuel Alizon and Mircea T. Sofonea

We have tried to take into account the criticisms and suggestions made by both reviewers. Every comment has been specifically replied to.

Comments to the Authors:

Reviewer #1:
Primary Comments

1) Tests for significance should be applied to determine if any differences between the Non-Markovian compartmental model and the three tested methods are statistically significant. The null hypotheses driving the comparisons should also be explicitly stated. * Additionally, the significance level utilized in the study should be presented in the "Materials and methods" section

Response: We have updated our Figures 6 and 7 to make comparisons of the Non-Markovian compartmental model to the three considered baselines with fully disclosure of the statistical significance. Explanations of the statistical testing procedure including null hypotheses are provided in the methods section as well as the Supplemental materials.

2) The study is not properly situated within the literature.
2.1. Similar to the supporting arguments that forecasting differences occur at local versus national interventions, there is also evidence that forecasts have statistically significant differences at varied geographic scale for COVID-19. Some suggestions follow: Barría-Sandoval, C., Ferreira, G., Benz-Parra, K., & López-Flores, P. (2021). Prediction of confirmed cases of and deaths caused by COVID-19 in Chile through time series techniques: A comparative study. PLOS One, 16(4), e0245414. Gecili, E., Ziady, A., & Szczesniak, R. D. (2021). Forecasting COVID-19 confirmed cases, deaths and recoveries: Revisiting established time series modeling through novel applications for the USA and Italy. PLOS One, 16(1), e0244173. Lynch, C. J., & Gore, R. (2021). Short-range forecasting of COVID-19 during early onset at county, health district, and state geographic levels using seven methods: Comparative forecasting study. Journal of Medical Internet Research, 23(3), e24925.

Response: We have included your suggestions in the introduction to emphasize the added

value of local forecasts for COVID-19.

2.2. The use of Median as a measure of central tendency for the metrics seems appropriate. However, this should be supported with relevant references. Recommend adding references to the "Standard metrics" section that discusses the use of median for testing for statistically significant differences across forecasts. A general example and a COVID-context example at different geographic resolution levels are provided: Lynch, C. J., & Gore, R. (2021). Application of one-, three-, and seven-day forecasts during early onset on the COVID-19 epidemic dataset using moving average, autoregressive, autoregressive moving average, autoregressive integrated moving average, and naïve forecasting methods. Data in Brief, 35, 106759. Hyndman, R. J., & Athanasopoulos, G. (2018). Forecasting: principles and practice. OTexts.

Response: While I am familiar with Hyndman's textbook, I am unaware of any discussion about using median summary statistics for forecast evaluation (he is more known as a Mean Absolute Scaled Error advocate). However 3 more citations have been added to the Standard metrics section (including your first suggestion) that utilize either median AE or median relative WIS for COVID-19 forecast applications.

3) The "Methods" section requires some additional information. Specific points follow:
3.1. With respect to "outlier errors" mentioned in section 1.6 "Standard metrics", please define how outliers are calculated within the context of this study.

Response: We are somewhat confused by comment because it is unclear why outliers would even need to be calculated in this context. They are self-evident and their presence is the motivation for using a robust statistic like the median over the mean. We referenced an entire part of a figure to depict the "exaggerated errors" at the wave peaks for national forecasts (note that some regions are even more extreme). To circumvent any confusion we now call these errors "abnormally large" or "exaggerated" instead of outliers.

4) The "Abstract" mentions that the provided model both performs better and worse than existing models for various scenarios. The testing metric, i.e., Absolute Error, Weighted Interval Score, etc., should be provided as well along with their corresponding sample sizes and p-values.

Response: We want to emphasize that that this evaluation is completely exploratory in nature (e.g. Exploratory Data Analysis by John Tukey) and not confirmatory. There is no hypothesis formulated a priori that is tested with an experiment and statistically assessed with a single p-value. The "experiment" was developing a real-time forecaster that was actually deployed during the pandemic and now we are trying to benchmark

and communicate weak and strong points of the surveillance system. This all said, even if we only consider AE, our simplified forecast evaluation depicts 2 forecast horizons, 14 geographical units and a minimum of 3 baseline comparisons, which produces 84 p-values. The updated manuscript includes a total of 168 p-values in the main text and the Supplementary information includes 504 for AE alone. While including p-values and sample sizes in the abstract may make sense for confirmatory analyses where most people only want to read the abstract (e.g. for a clinical trial), it does not seem to add value to do that here. We do concede to the overall point that saying that a model performs better or worse needs to be quantitatively justified. The abstract has been changed to describe the distribution of statistically significant performance improvements over regions.

5) This is a comparative study, and yet, the presentation of results is the shortest section in the article. This section lacks any quantitative support beyond pointing at the figures. Many generic claims are made such as "...shows improvement relative to other baselines..." without providing any statistical support to backup the claim. This section needs to be expanded to provide an in-depth comparison of the forecasting techniques, to include the presentation of tests for significant differences between techniques.

Response: The Results section has been expanded and includes direct comparisons of COVIDici to the baseline model performance including p-values.

6) A "Limitations" section should be added to the text prior to the "Conclusion". This section should discuss the limitations of the study, the generalizability of the results, and validity concerns.

Response: A limitations section for the study and model has been added before the conclusion.

Additional Comments – In order of appearance: *) Common issue across many of the figures, text is small and difficult to read and difficult to related points to their corresponding values on the y-axis. Please assess all figures for readability.

Response: The size of the text in the figures may be more the responsibility of the typesetter. We are just following the guidelines of the journal. We have tried to address your concerns in all new figures and done our best to improve the old ones.

Reviewer #2:

Major Comments:

1. Although many of the methods used to perform this retrospective eval-

uation have already been published, the manuscript lacks detail about model evaluation and fitting here in this manuscript. This needs to be addressed with a revision to section 1.3 Calculation. The statement, "Details on the inferred parameters, their prior values and distributions are provided in [16]," is not adequate, especially since the model presented in Figure 1 [16] does not include vaccinated susceptibles. It would be helpful to have a table of mathematical notation and parameters in the supplement describing their definitions and how each of these were fitted, similar to [16]. More detail about the calculation and maximization of the Poisson likelihood to get the expectation and the variance of the infection-to-hospitalization delay using MCMC also needs to be added to the supplemental, as well as a justification for a Poisson model. The algorithms and software used for model evaluation and fitting should also be described and cited.

Response: As recommended by the reviewer, whom we thank very much for her/his pertinent suggestions, we have added a paragraph in section 1.3 Calculation which details the inference procedure and justifying the Poissonian likelihood.

Furthermore, in the Supplementary material, we have added 5 tables defining the main model variables, the inferred key parameters (detailing the priors used at national and sub-national level), the constrained parameters, the probability distributions involved and related bibliographic or database sources, as well as additional notations useful for understanding the parameterization of the model as well as the demonstration we have added, justifying the adjustment of the current transmission factor.

Moreover, we have mentioned the software and algorithms used in the Materials and methods section.

2. The forecasting methods are qualitatively compared in Figure 3, and the authors use several metrics (e.g. AC, ECR, WIS, Brier score) to quantify forecasting errors to compare models. Comparisons in forecasting error are shown in Figure 4, and the authors claim that COVIDici is a top choice for four-week horizons. The forecasts for each model should be compared statistically (i.e. with measurements of uncertainty such as credible intervals for each curve) in order to determine when COVIDici outperforms other methods. If possible, it would also be valuable if the authors could quantify the expected impacts of the forecasting errors. For example, does the difference in percentage of incorrect forecasts for ICU overload between each model in Figure 5A at four weeks translate into substantial procedural differences in hospitals? Stronger evidence is needed to claim that COVIDici is a better forecasting model over longer time scales, and this should be updated in the Results and Discussion.

Response: Confidence intervals based on a non-parametric bootstrap have been added to all Figures. The only exception is for binary metrics that have direct interpretation such as % of incorrect forecasts that is obscured when making the graph too busy. Note

that CIs are included for the relative binary metrics so model comparison can be clearly viewed. We also added new forest plots with confidence intervals for absolute error and WIS. It seems quite interesting but possibly out of scope for this manuscript to examine how forecast errors translate into procedural differences in hospitals. That would require exhaustive within-hospital modelling such as in https://doi.org/10.1007/s10729-021-09548-2, which doesn't seem very straightforward to integrate into this study.

Regarding stronger evidence that COVIDici showed better forecast performance at longer horizons, of the 10 statistically significant improvements (90% level) in median AE that were detected at the 4-week horizon all of them favored COVIDici. Under the null the p values should be uniformly distribution. We have included a comment that this is strong indication that the significant tests are not merely the result of a multiple testing problem. Only one region showed COVIDici statistically outperforming the ensemble ETS+ARIMA which was a pair that were for the most part undistinguishable statistically. Nonetheless, this shows a clear albeit modest improvement at the 4 week horizon. The abstract, results and discussion section have been changed to reflect this argument.

3. Inline 263 the authors state, "Figure 5C breaks these metrics down by region which supports similar conclusions." There is so much overlap in the forecast results by region in 5C, that I disagree that any conclusion can be drawn here. The authors need to further explain and justify this statement.

Response: This figure has been redone completely when including confidence intervals. It was decided to drop the breakdown by region (previously 5C) for binary metrics because it added too much "noise" to an already busy figure. Thus, this comment no longer needs to be addressed.

Minor comments:

4 – "has led to" – change adopted

Figure 3B – the bold vertical text is hard to impossible to read. This should be fixed. – We will keep this in mind but this is an issue for the typesetter. When the manuscript is printed it is clearly legible, but maybe not in the format you saw.

Figure 3C and Figure 1 in Supplemental should have X- and Y-axes with units. – Adding units would add no value to the graph as an abstract artistic schematic. We added a label that it is indeed "artistic" (like a supply and demand curve) because it is not based on any real data.

249 – Please refer to a figure here. – Restructuring the results sections has made this comment irrelevant.

Figure 4A – Why does WIS have a double asterisk in this panel? – It was a typo but the figures have been redone so this is no longer relevant.

Figure 5C – Y-axis needs a label. – Figure 5C has been removed and the entire figure has been updated to include confidence intervals for relative binary metrics.

Supplemental – In the Calculation section, the authors state, "As a result, we fitted a skewed normal distribution when the point forecast value was greater than 6 (daily events) or a log-normal distribution when the point was less than 6 (i.e. close to zero) using quantile matching." Please give a reason or citation for why 6 is your cutoff value and why you change the distribution as these values change. – Skewed normal distributions can take negative values whereas log-normal distributions cannot. The fit of the distribution was done visually by looking at several examples. Make 6 the cutoff balanced fit and the desire to avoid negative quantile forecasts. An explanation of this has been provided in the Supplementary materials.

We thank the reviewers for their valuable comments which clearly improved the manuscript greatly. We hope that it is now suitable for publication and we thank the editors for their contribution.

Alexander Massey and the rest of the ETE Modelling Team
