# Supplementary material for: Real-time forecasting of COVID-19-related hospital strain in France using a non-Markovian mechanistic model
Source: PLoS Comput Biol. 2024 May 17;20(5):e1012124. doi: 10.1371/journal.pcbi.1012124 (PMC11139328; doi:10.1371/journal.pcbi.1012124)
Supplement: Attachment — Submitted filename: FInal_Response_to_reviewersPLOScompbiol.pdf [file pcbi.1012124.s003.pdf]

Manuscript ID: PCOMPBIOL-D-23-01056

Real-time forecasting of COVID-19-related hospital strain in France using a non-Markovian mechanistic model

by Alexander Massey, Corentin Boennec, Claudia Ximena Restrepo-Ortiz, Christophe Blanchet, Samuel Alizon and Mircea T. Sofonea

We once again thank the reviewers for their thoughtful feedback and are happy that they both pleased with the revised version of this manuscript. We respond specifically to their final comments/suggestions below.

Comments to the Authors:

Reviewer #1:
Minor suggestion:

It may be worthwhile to remind the reader at the start of the Limitations section that these limitations should be interpreted within the perspective that COVIDici was being utilized and updated during the pandemic. Limitation 2 may be a very enlightening point for readers in helping to understand the importance of these types of studies in evaluating the effectiveness of techniques that have been applied in practice during a practical setting to help in informing future practitioners in future global crises.

Response: We incorporated into the Limitations section a short reminder that there is an important practical context for how a surveillance system is implemented in real-time that does not have the benefit of hindsight.

Reviewer #2:
Major Comment:
The authors clearly state this is retrospective study and should be evaluated in the context of decreased surveillance. These are important points and I appreciate the authors making these clear. However, we are mostly past the pandemic, and it would be helpful for the authors to explain how their analysis can help us prepare for the future. I think it would add impact to the paper if the authors could add information about how these results can more generally inform our ability to make forecasts for hospital strain during disease outbreaks or to prepare for the next pandemic. The only place the authors really do this is in the last sentence in the Author summary, but this could be strengthened. I think statements about the importance of this work to the future need for this type of forecasting should be added to the abstract,

the last sentence of the introduction in lines 59-60, and the last paragraph of the conclusion.

Response: We have added statements emphasising that we recommend non-Markovian compartmental models for surveillance of future pandemics.

Minor comments:

Revise the first sentence of the Author Summary to: "The US and European Covid-19 Forecast Hubs focus on metrics such as deaths, new cases, and hospital admissions, but do not offer measurements of hospital strain like critical care bed occupancy..." – change adopted.

Line 72 – Please provide the doi associated with the Zenodo repository. – The DOI for this repository is clearly indicated in the references which is linked by the citation.

Line 150 – Revise the sentence in line 150 to: "The random variable can reasonably be assumed to be Poisson-distributed. This assumption works well for small admission numbers because the population sizes of the investigated locations are large while the daily individual probability of being admitted in an ICU for COVID-19 is small." – change adopted

We again thank the reviewers for their valuable comments which continue to improve the manuscript. We hope that this final version is now suitable for publication and we thank the editors for their contribution.

Dr. Alexander Massey on behalf of the co-authors
